# Ruxolitinib Associated Tuberculosis Presenting as a Neck Lump

**DOI:** 10.1155/2015/284168

**Published:** 2015-12-15

**Authors:** Eamon Shamil, David Cunningham, Billy L. K. Wong, Piyush Jani

**Affiliations:** ENT Department, Addenbrooke's Hospital, Cambridge University Hospitals NHS Foundation Trust, Hills Road, Cambridge CB2 0QQ, UK

## Abstract

Tuberculosis is an opportunistic infection with protean clinical manifestations. We describe a case of Ruxolitinib induced miliary tuberculosis presenting as a neck lump. A 78-year-old female presented with a two-month history of right-sided neck lump associated with fever, night sweats, and significant weight loss. She had a past medical history that included myelofibrosis, being treated with Ruxolitinib. Examination demonstrated 4 × 4 cm right-sided cervical lymphadenopathy. A chest radiograph showed extensive shadowing in both lungs. CT scan demonstrated perilymphatic nodes in addition to the cervical mass. An ultrasound-guided biopsy of a cervical lymph node demonstrated confirmed* Mycobacterium tuberculosis* infection. It was hypothesized that use of Ruxolitinib through its selective inhibition of Janus-activated kinases 1 and 2 resulted in immunosuppression and miliary tuberculosis in this patient. The medication was stopped and a 12-month regime of antituberculosis therapy commenced. She remained well at one-year follow-up with resolution of lung involvement. Clinicians should consider tuberculosis as a differential diagnosis for patients presenting with a neck lump, particularly in those taking immunosuppressant medication such as Ruxolitinib. A multidisciplinary approach is needed to promptly treat the tuberculosis and consider discontinuation of Ruxolitinib.

## 1. Introduction

Myelofibrosis is a subtype of myeloproliferative neoplasms (MPN), characterised histologically by megakaryocyte proliferation, dysregulation of cytokines, and subsequent fibrosis of bone marrow [1]. Symptomatically, patients present with anaemia, splenomegaly, and constitutional symptoms such as fatigue, night sweats, weight loss, pruritus, and bone pain. Ruxolitinib is a novel therapeutic agent developed for the treatment of myelofibrosis. It is a selective inhibitor of JAK1 and JAK2 and has significant positive effect on constitutional symptoms and splenomegaly [1–3]. Two cases of tuberculosis in patients taking Ruxolitinib have been noted in a key phase III clinical trial [3] and several cases of opportunistic infections have been reported in the literature [4–10].

We report the first case of disseminated tuberculosis presenting with a neck lump following the use of Ruxolitinib. Multidisciplinary decision should be taken to promptly treat the tuberculosis and consider discontinuation of Ruxolitinib.

## 2. Case

A 78-year-old female presented to the Ear, Nose and Throat Clinic with a two-month history of right-sided neck lump having been referred by her General Practitioner. Upon further screening, it became apparent that she had been experiencing associated fever, night sweats, and significant weight loss. She had previously been diagnosed with myelofibrosis and her symptoms were well controlled through use of Ruxolitinib. Examination revealed 4 × 4 cm right-sided lymphadenopathy in levels 3 and 4 of the neck which appeared necrotic and was discharging pus. Examination of her oral cavity and oropharynx was normal and fibreoptic nasendoscopy revealed no abnormalities within her postnasal space, pharynx, and larynx. A host of investigations were carried out to narrow the list of differential diagnoses. Computed tomography (CT) scan of the neck and chest showed a large right cervical mass with no other abnormalities within the neck. There was no evidence of metastatic disease; however, perilymphatic nodules were noted. The differential diagnoses at this stage were sarcoidosis, tuberculosis, or malignancy. Definitive diagnosis required histological and microbiological analysis of the neck lump. An ultrasound-guided biopsy of a cervical lymph node did not show any malignant cells but the presence of mycobacterium was identified on Ziehl–Neelsen staining. Subsequent microbiology culture confirmed* Mycobacterium tuberculosis* infection. A chest radiograph also showed extensive shadowing in both lungs consistent with miliary tuberculosis.

Ruxolitinib was discontinued instantly and the patient was started on a 12-month regime of antituberculosis therapy comprising Rifampicin, Isoniazid, Pyrazinamide, and Ethambutol. She remained well at one-year follow-up with resolution of miliary nodules throughout her lungs.

## 3. Discussion

The underlying pathogenesis of myelofibrosis is still not fully understood. A mutation in the tyrosine-protein kinase Janus-activated kinase 2 (JAK2) is thought to result in upregulation of proinflammatory cytokines [2, 4, 5]. Ruxolitinib works by inhibiting JAK1 and JAK2, resulting in immunosuppression. Specifically, Ruxolitinib depresses the T-helper cell type 1 (Th 1) response and downregulates cytokines such as interleukin-1, interleukin-6, interferon-*γ*, and tumour necrosis factor-*α* [11, 12]. Recently it has been shown that Ruxolitinib impairs dendritic cell development and function, including production of interleukin-12 by dendritic cells [2, 4].

Clinical trials have demonstrated a reduction in splenomegaly and improvements in myelofibrosis symptoms when compared with conventional treatment [3]. However, the effects Ruxolitinib has on one's immune system can be profound and may underlie the pathogenesis of opportunistic infections associated with the medication. Sporadic cases of progressive multifocal leukoencephalopathy, toxoplasmosis retinitis,* Cryptococcus neoformans* pneumonitis, herpes zoster, and reactivation of hepatitis B have been documented in the literature [3, 6–9]. Four such cases of Ruxolitinib associated tuberculosis have been described as case reports in the literature to date [1, 13–15] and two cases noted in a phase III trial [3]. Of the four case reports, there were two cases of disseminated tuberculosis [1, 13], one case of extrapulmonary [14] and one case of pulmonary tuberculosis [15], respectively. All patients presented with fever but two had constitutional symptoms of tuberculosis such as weight loss, fever, night sweats, and anorexia. Two had lymphadenopathy in axillary and inguinal regions, respectively [14, 15]. The patient that we present in this paper is the only case presenting with a neck lump. There is speculation whether these cases were primary or reactivation of latent tuberculosis. Only 5% of individuals develop primary tuberculosis following contact with individuals with active tuberculosis infections. 95% respond to* M. tuberculosis* by encasing the bacteria in granulomas, and the infection enters a latent phase. However, the use of biologics such as Ruxolitinib can cause reactivation of the infection [15]. The longest duration of Ruxolitinib treatment before the onset of tuberculosis symptoms in this cohort was four months while the shortest was two months. The close chronological relationship between the administration of Ruxolitinib therapy and development of tuberculosis-related fever makes reactivation of tuberculosis a more likely diagnosis although two of the patients did not have any history of past tuberculosis infection.

Colomba et al. [1] did not describe if Ruxolitinib was discontinued in their case, but all other papers including the present case discontinued Ruxolitinib treatment on patient presentation. Standard therapy for tuberculosis comprising Isoniazid, Rifampicin, Pyrazinamide, and Ethambutol was immediately commenced on diagnosis for all cases. Chen et al. [15] and Colomba et al. [1] did not specify the duration of the tuberculosis treatment but Palandri et al. [14] treated their patient for six months whereas both Hopman et al. [13] and the current paper instituted tuberculosis therapy for about 12 months due to miliary TB.

Despite the risk of further immunosuppression, Palandri et al. [14] and Hopman et al. [13] restarted Ruxolitinib treatment for their patients due to significant relapse of myelofibrosis symptoms.

Interestingly, Palandri et al. [14] left their patient on long-term Isoniazid as prophylactic treatment, with no signs of tuberculosis reactivation. This was supported by Heine et al. [16] who suggested the use of antiviral and antibiotic prophylaxis for patients undergoing Ruxolitinib therapy. Provisional screening in tuberculosis endemic areas or if any risk factors for such an infection are present has also been suggested prior to starting this treatment [13, 14, 16].

Opportunistic infections occur in patients taking immunosuppressant medication such as Ruxolitinib. Clinicians should consider tuberculosis as a differential diagnosis in patients who present with cervical lymphadenopathy and also take Ruxolitinib.

After commencing Ruxolitinib, regular follow-up of patients is advised, especially for the first six months, to assess for the development of opportunistic infections such as tuberculosis. A multidisciplinary approach is required when assessing the risk versus benefit of starting and stopping medications that may predispose to infection. Multidisciplinary discussion should include the benefit of long-term prophylactic antituberculosis and antiviral therapy.
